# Outcome of acute kidney injury: how to make a difference?

**DOI:** 10.1186/s13613-021-00849-x

**Published:** 2021-04-15

**Authors:** Matthieu Jamme, Matthieu Legrand, Guillaume Geri

**Affiliations:** 1 Service de Réanimation, Hôpital de Poissy, CHI Poissy Saint Germain, 10 rue du champ Gaillard, 78300 Poissy, France; 2 INSERM UMR 1018, Equipe Epidémiologie clinique, CESP, Villejuif, France; 3 Université Paris Saclay, UFR Simone Veil - Santé, Montigny-Le-Bretonneux, France; 4 Department of Anesthesia and Perioperative Care, University of California, San Francisco, USA; 5 Service de Médecine Intensive Réanimation, Hôpital Ambroise Paré, AP-HP, Boulogne Billancourt, France

**Keywords:** Acute kidney injury, Long-term outcome, Chronic kidney disease, Intensive care

## Abstract

**Background:**

Acute kidney injury (AKI) is one of the most frequent organ failure encountered among intensive care unit patients. In addition to the well-known immediate complications (hydroelectrolytic disorders, hypervolemia, drug overdose), the occurrence of long-term complications and/or chronic comorbidities related to AKI has long been underestimated. The aim of this manuscript is to briefly review the short- and long-term consequences of AKI and discuss strategies likely to improve outcome of AKI.

**Main body:**

We reviewed the literature, focusing on the consequences of AKI in all its aspects and the management of AKI. We addressed the importance of clinical management for improving outcomes AKI. Finally, we have also proposed candidate future strategies and management perspectives.

**Conclusion:**

AKI must be considered as a systemic disease. Due to its short- and long-term impact, measures to prevent AKI and limit the consequences of AKI are expected to improve global outcomes of patients suffering from critical illnesses.

## Introduction

Acute kidney injury (AKI) is one of the most frequent organ failure encountered in intensive care units (ICU). Since his first definition by Homer W. Smith in the fifties [1], more than 30 different definitions have been used, leading to a sizeable epidemiological heterogeneity [2] with incidence ranging from 5 [3] to 25% [4].

Since 2004, three definitions, based on serum creatinine (SCr) and urine output, respectively: RIFLE [5], AKIN [6], and the actual KDIGO classification [7] have been proposed allowing homogenization of AKI definition as well as epidemiological association between AKI and chronic kidney disease (CKD). Based on the most recent KDIGO definition, AKI occurs in more than a third of ICU patients [8, 9].

## Why physicians should worry about AKI?

Occurrence of AKI represents a sharp prognostic turn for patients by affecting both short- and long-term prognosis.

### AKI and global (short and long term) prognosis

The multinational EPI-AKI study has highlighted that AKI was associated with short-term mortality in a severity-dependent manner (OR = 2.19 [1.44–3.35], 3.88 [2.42–6.21] and 7.18 [5.13–10.04] for KDIGO stage 1, 2 and 3, respectively) [8]. All subgroups of ICU patients seemed to be affected [10–15]. A poor short- and mid-term outcome was also observed in patients with sub-clinical AKI (defined by positive biomarkers of kidney injury but not meeting the current definition of AKI) [16].

Moreover, AKI has been repeatedly associated with poor long-term outcomes [17]. In a large study reporting 1-year outcome of more than 16,000 patients discharged alive from the hospital and who suffered AKI in ICU, five profiles were identified according to the renal status during ICU and hospital stay: patients with early (< 7 days from admission) or late (> 7 days) sustained recovery, relapse with (relapse no recovery) or without altered renal function at hospital discharge (relapse recovery) and sustained renal failure [18]. Patients with altered renal function at hospital discharge (never reversed or relapse no recovery) had the worst outcome. Interestingly, even patients who apparently recovered from AKI at ICU discharge (based on serum creatinine) but with positive biomarkers of kidney injury had a higher risk of death during the year following ICU discharge. Once again, this suggests that beyond the impact of decrease renal function, kidney damage impacts long-term outcomes [19].

### AKI and chronic kidney disease (CKD)

The end of 2010s has been marked by the publication of several studies that highlighted an association between AKI and subsequent CKD occurrence. Wald et al. have compared 3,769 to 13,598 matched patients treated or not treated with renal replacement therapy (RRT) in ICU and observed a higher incidence of end-stage renal disease with RRT (2.63 vs. 0.91/100 patient-years, hazard ratio = 3.23 [2.70–3.86]) [20]. In a Swedish national cohort of 97,782 ICU patients, Rimes-Stigare et al. have reported that patients who suffered de novo AKI had an increased risk of CKD (adjusted incidence rate ratio = 7.6 [95%CI 5.5–10.4]) and end-stage renal disease (ESRD) (adjusted incidence rate ratio = 22.5 [95% CI 12.9–39.1]) compared to patients without AKI during their ICU stay [21]. The same group identified that CKD at ICU admission and severity of AKI was associated with ESRD in 1-year survivors [22]. Similar observations were made in specific subgroups like elderly [23], pediatric [24], diabetic [25], post-cardiac surgery [26], or resuscitated cardiac arrest patients [27].

Interestingly, patients who fully recover at hospital discharge remain at risk of CKD 1 year afterwards, particularly in the case of subsequent episodes of AKI during the ICU stay [18]. Of note, all these studies were retrospective or provided results from electronic administrative datasets with significant risk of bias. A major recent prospective study clarified the association between AKI and CKD. In the Assessment, Serial Evaluation, and Subsequent Sequelae IN Acute Kidney Injury (ASSESS-AKI) Study, a multicenter prospective study comparing 769 patients with or without AKI, the authors observed that an increased urinary albumine-to-creatinine (ACR) ratio at 3 months after discharge was the most predictive biomarker of kidney disease progression (HR = 1.25 [1.10–1.43] per doubling of urine ACR, P < 0.001). Interestingly, in multivariable analysis, AKI occurrence was not associated with kidney disease progression [28]. However, we should not neglect the importance of AKI in the evaluation of renal prognosis regards to the sensitivity analyses performed using the urine protein-to-creatinine ratio instead of urine ACR. In that case, AKI became strongly associated (HR = 2.53 [1.21–5.25], *p* = 0.01) with kidney disease progression. Moreover, C statistic used to discriminate risk of poor renal outcome was better in the latter (0.84 vs 0.79).

Our current understanding is that an acute episode leaves an imprint (which is expected to be, biologically, of epigenetic nature) able to promote renal fibrosis [29, 30]. However, mechanisms leading to CKD in this context are not yet fully understood.

### AKI and long-term cardiovascular risk

In several extensive large cohort studies, AKI has been associated with an increased risk of a cardiovascular events [31–33], especially heart failure. In a recently published meta-analysis, Otudayo et al. reported a 58%, 40%, and 15% increased risk of heart failure, myocardial infarction, and stroke, respectively [34]. Mechanisms leading to cardiovascular events are not elucidated so far. Accelerated atherosclerosis might be a contributing factor [35]. In a translational study performed in 968 adults undergoing cardiac surgery, patients with clinical AKI, and elevated cardiac injury biomarkers at day 1–3 were strongly associated with long-term cardiovascular events. Other mechanisms involving mitochondrial dysregulation have also been suggested. Sumida et al. showed increased cardiomyocyte apoptosis and cardiac dysfunction after renal ischemia reperfusion in a mouse model. The authors also observed a significant increase of mitochondrial fragmentation in cardiomyocytes with an accumulation of an unique fission regulation protein: Drp1 [36].

In contrast, urinary kidney injury biomarkers at day 1–3 were not associated with outcome [37]. These results suggested that AKI was indicative of cardiovascular stress rather than an independent renal pathway. However, an association between the occurrence of cardiovascular events and AKI remaining after adjustment for cardiovascular risk factors and preclinical data argue for a direct impact of AKI on cardiovascular damage [38].

This hypothesis has also been demonstrated in a murine experimental work highlighting the role of the galectin-3 pathway [39]. Prud'homme et al. have evidenced AKI increased galectin-3 expression, which induced cardiac inflammation with macrophage infiltration and cardiac fibrosis resulting in cardiac dysfunction.

## The three critical stages of renal management: before, during, and after AKI (Fig. 1)

**Fig. 1:**
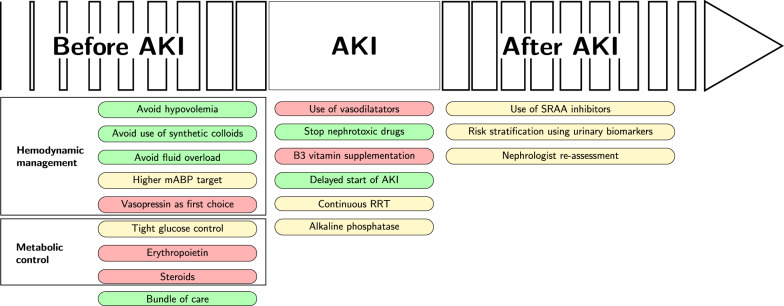
The three steps of renal management. Green, yellow and red boxes represented interventions with, respectively, surely, possibly and insufficient level of evidence of benefit. AKI, acute kidney injury; mABP, mean arterial blood pressure; RRT, renal replacement therapy; SRAA, renin angiotensin aldosterone system

### Before AKI: prevent AKI to occur

The cornerstone of AKI prevention in ICU patients is the management of hemodynamics, including appropriate volume of fluids, the choice of fluids and of vasoactive drugs. Even if the pathogenesis of AKI in ICU patients may rely on different mechanisms [8, 40], hemodynamic optimization appears essential to prevent alterations of renal blood flow (RBF) [41].

### Hemodynamic management

Appropriate volume replacement should be performed as early as possible while keeping in mind that fluid overload was reported to be associated with poor prognosis in AKI patients [42, 43]. This apparent antagonist observation probably highlights the higher severity of AKI patients requiring higher volume of fluids and the critical role of the timing of fluid administration during the course of critical illness. Since the first alarming publications about the nephrotoxicity of artificial colloids [44, 45], although probably less harmful in less severe patients [46], crystalloids are the solutions of choice for ICU patients [47, 48]. Indirect and observational results suggested better renal outcomes with so-called balanced solutes [49, 50]. This could have been explained by the deleterious effects of hyperchloremia acidosis induced by a highly concentrated solution in chlorine [51]. If these observations could not be verified by the randomized clinical trial SPLIT (relative risk for AKI occurrence within 90 days = 1.04 [0.80–1.36], *p* = 0.77), the absence of sample size calculation added to the non-control administration of the solutes before admission to ICU limited the interpretation of this results [52]. In the Isotonic Solutions and Major Adverse Renal Events Trial (SMART) in ICU and non-ICU patients, a protective renal effect favoring the use of balanced solution with an absolute reduction in the risk of major adverse kidney events by 1.1 [1.092–1.107] % for ICU patients and 0.9 [0.889–0.911] % for non-ICU patients [53, 54] was observed. However, it is important to note that the frailty index calculated for the SMART studies, which is a complementary means to the p value for the interpretation of the results of the clinical trials, was very low. This observation suggesting a low robustness of the results [55]. But frailty index can also be interpreted as the reflection of a consistent choice of the size of the population studied for the size of the effect observed. Finally, its use is recently debated because it has been proven to lack the ability of the frailty index to quantify deviations from a model's null assumptions [56]. While the case of balanced crystalloids vs normal saline is not closed, accumulating evidence strongly suggest that (1) normal saline is not superior to balanced solution and (2) balanced solution are likely to be superior to normal saline in acutely and critically ill patient. Pending ongoing trials, this justifies in our view the use of balanced solutions as first line fluids in ICU patients.

Besides the choice of solute, the concept of the optimal mean arterial pressure target has been advocated for a long time. In the EPI-AKI study, factors associated with AKI included a past medical history of hypertension or shock at ICU admission, with higher ssimplified acute physiology score 3 [8]. These results are in line with those of the SEPSIS-PAM trial [57]. SEPSIS-PAM was a randomized controlled trial (RCT) targeting a mean arterial pressure of either 65 or 85 mmHg. It evidenced a significantly lower proportion of severe AKI and rate of renal replacement therapy in patients with chronic hypertension in the higher blood pressure group (31 vs. 42%, *p* = 0.04) [57]. This relationship had been demonstrated in physiological studies which strongly suggested that glomerular filtration rate (GFR) and RBF can vary widely across mean arterial pressure (mABP) ranges, but, however, the impact of raising mABP on renal hemodynamic varies on an individual basis [58]. In the 65 trial, a strategy of permissive hypotension strategy vs. usual care in patients aged 65 years or older and admitted to ICU for vasodilatory hypotension was tested. No difference was observed with respect to the incidence of RRT, including among the subgroup of patient with a history of hypertension [59]. However, such a lack of difference should be interpreted with caution due to the small difference of mABP levels between groups (respectively mABP of 67 [64–70] mmHg and mABP of 73 [69–76] mmHg). In another ICU population, renal adverse events were less frequently observed in the high target patients (4 vs. 9%, *p* = 0.002) in an RCT including patients admitted to the ICU for acute intracerebral hemorrhage [60]. Taken together, these findings do not support yet a wide use of higher mABP targets in patients with shock to protect the kidney. However, physiological studies strongly suggest that glomerular filtration rate and renal blood flow can vary widely across mABP ranges and the impact of raising mABP on renal hemodynamic varies on an individual basis [58].

During shock, it has been well demonstrated that a decrease in blood pressure below the limit of renal self-regulatory capacity lead to an almost linear drop in RBF. While norepinephrine remains the first-choice vasopressor to maintain arterial perfusion, its direct effects on RBF remains controversial. On one hand, norepinephrine has been shown to decrease RBF in healthy volunteers and its nephrotoxic impact is frequently used in fundamental research on animal models to promote AKI [61, 62]. On the other hand, in distributive shock, the use of norepinephrine restores RBF [58, 63]. Vasopressin has been suggested to improve renal outcomes in preliminary reports. However, vasopressin has not yet been shown superior to norepinephrine in preventing AKI in ICU patients [64–66].

### Improve the oxygen supply/need balance

Numerous other procedures aiming to improve intra-renal perfusion or oxygenation have been evaluated, including renal vasodilators, control of renal hypercatabolism, anti-inflammatory, and antioxidants drugs. Among them, dopamine is undoubtedly the most extensively studied one. Its administration at low doses (< 5 µg/kg/min) inducing special in dopaminergic and β-adrenergic effect and therefore causes renal vasodilatation. However, despite intensive research for more than 30 years, date remain largely inconclusive to prevent occurrence of AKI [67]. Other vasodilators agents, like fenoldopam, B-type natriuretic peptide, and levosimendan, have failed to show any renal benefit [68–71]. Erythropoietin, steroids, tight glucose control, and numerous metabolic interventions have also been used to prevent kidney damage in various conditions. Except for the control of blood glucose level for which conflicting results have been obtained [72, 73], no renal benefit has been observed with these metabolic interventions as well [74–76].

### Bundles

**Fig. 2:**
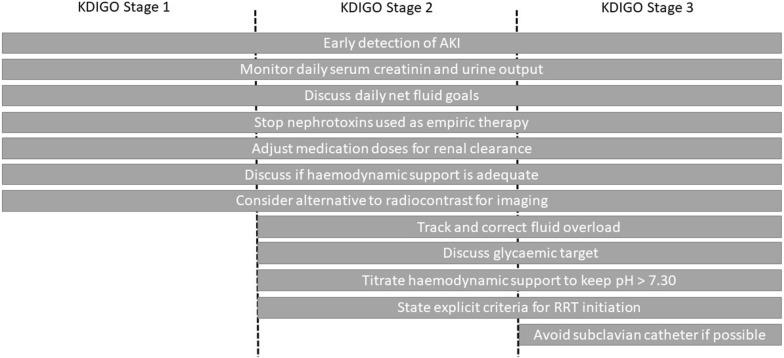
Acute kidney injury bundles of care (derived from the KDIGO AKI management guidelines). Grey boxes indicate action to establish according to KDIGO severity stages. AKI, acute kidney injury; RRT, renal replacement therapy

Beyond a single intervention, "bundles" have been proposed to prevent AKI [77, 78]. Bundles are a small, straightforward set of evidence-based practices that have been proven to improve patient outcomes when performed collectively and reliably (Fig. 2). This seems to allow better recognition [79] and reduce the risk of AKI progression [80]. Implementation of bundles has been able to demonstrate a reduction in the incidence of AKI in specific settings such as nephrotoxic AKI or post-cardiac surgery [81, 82]. Whether implementing those bundles in general ICU population or in sepsis could prevent AKI is still unknown.

### During the AKI: improving early recovery from AKI

#### Activation of the PGC1α-NAD pathway

While no specific treatment of AKI is yet available, numerous advances in the understanding of the mechanisms leading to AKI in ischemic or septic conditions have been made over the last years. Among them, the PPAR Gamma Coactivator 1 alpha Nicotinamide Adenine Dinucleotide (PGC1α-NAD) pathway is one of the most promising targets for AKI. As renal proximal tubular cells are one of the most energy or ATP demanding cells in the body, they are very dependent on mitochondrial function. An increase in the expression of PGC1α in renal epithelial cells subjected to ischemic stress was found protective, with a rise of NAD + [83, 84]. Furthermore, decrease expression of PGC1α was observed on human kidney biopsies in patients with AKI [84]. PPAR agonists have been proposed to prevent AKI induced by cisplatin or ischemia–reperfusion [85, 86]. The first class tested was fibrates, with mixed results [85, 86]. Another approach was to increase the oxidation of fatty acid (AF) by improving the transport of AF in the mitochondrial matrix using association with carnitine and an activator of the carnitine palmitoyl-transferase 1 also called enzyme of the carnitine shuttle [87]. However, these are essentially preclinical data with no evaluation in patients. Nicotinamide (Nam), the amine form of Vitamin B3 (niacin), was identified as a potential stimulator of the production of NAD + [88]. After promising preclinical experiments, the administration of Nam was evaluated in the prevention of postoperative AKI in cardiac surgery in a single center trial with encouraging results [89]. In this phase 1 pilot study, 37 patients after cardiac surgery were randomly assigned in three groups: placebo, nicotinamide 1 g per day and 3 g per day. The areas under the curve of all longitudinal SCr measured after randomization were higher in placebo group vs patients received nicotinamide supplementation. While these results deserve to be reassessed in larger samples with more suitable outcome, emerging data linking the NAD + equilibrium to AKI resistance opens a new exciting chapter in AKI research [88, 90].

### Renal replacement therapy: the right time to the right patient

The modality and the timing of initiation of RRT impact renal outcome. Concerning the modalities, it has been historically suggested that continuous techniques is associated with better hemodynamic stability [91]. Continuous RRT (CRRT) appear to result into fewer hypotension episodes during RRT sessions, allowing better renal perfusion, and, therefore, better recovery of renal function [92], potentially due to lower ultrafiltration rate and lower osmotic shifts compared to IHD [93]. After identifying 6,627 patients treated by RRT in ICU and survivors at day 90, Wald et al. were able to compare 2,004 patients treated with CRRT with 2,004 patients treated with intermittent hemodialysis (IHD) using a propensity score matching. Patients treated with IHD vs. CRRT were at higher risk of ESRD at 90 days (8.2 per 100 patient-years vs. 6.5 per 100 patient-year; HR = 0.75 [0.65–0.87]) [94]. However, these results were not confirmed in a subsequent study, which included 638 patients admitted to a single tertiary care academic medical center for 8 years and treated with RRT. After applying a conditional logistic regression model stratified by propensity score for CRRT, there was no significant higher risk of dialysis dependence at day 90 (OR = 1.19 [0.91–1.55] for CRRT, *p* = 0.20) and day 365 (OR = 0.93 [0.72–1.20] for CRRT, *p* = 0.55). Even if a difference favoring CRRT at day 90 was observed (186/244 (76.2%) for CRRT patients vs. 66/101 (65.3%) for IHD patients, *p* = 0.05), this association did not remain significant at day 365 [95]. Exploration of the French electronic health record revealed an association between the use of IHD and the risk of developing CKD among ICU patients [96]. The KDIGO guidelines suggest the use of CRRT for patients with unstable hemodynamics but with moderate level of evidence [7], since available RCTs were not designed to address the impact on renal outcome [97]. While the timing of renal replacement therapy does not affect survival in critically ill patients [98–102], data suggest potential harm of a liberal use of RRT on renal recovery. No difference in renal recovery was observed at day 90 in both the ELAIN study (9/67 (13.4%) for the early group vs. 8/53 (15.1%) for the delayed group, *p* = 0.80) and the IDEAL-ICU trial (2/101 (2%) for the early group vs. 3/110 (3%) for the delayed group, *p* > 0.90) [99, 102]. Recently, a higher RRT dependence among survivors at day 90 was observed in the STARRT-AKI study (85/814 (10.4%) for the accelerated group vs. 49/815 (6.0%) for the standard group). Concerning long-term outcome, analysis from the extended 1 year follow-up of the ELAIN study suggested better prognosis of early initiation of RRT whether in terms of mortality (absolute difference − 19.6 (− 32; − 7.2) %, *p* < 0.01) or recover of renal function (absolute difference = − 34.8 (− 54.6; − 15) %, *p* = 0.001) [103].

### After AKI: Preventing the long-term consequences

#### The maladaptive repair concept and the evolution to the renal fibrosis

For a long time, the suspected renal lesion of AKI was acute tubular necrosis (ATN), otherwise described as transient with full recovery. It is now well established that that the repair after ATN is ultimately imperfect, culminating in the concept of "maladaptive repair." This "maladaptive repair" initiates fibrogenesis even when morphology and renal function have apparently returned to normal. Similarly, increasing evidence in kidney transplants suggests that ischemic episodes are connected to transplant fibrosis [104]. So far, four significant pathways have been identified to trigger fibrosis after an episode of transient AKI: (a) the epigenetic silencing of *RASAL1*, a proliferation inhibitor, in myofibroblasts; (b) the cell cycle arrest in G2/M in tubular epithelial cells (the G2/M phase is where the epithelial cell function is closer to a mesenchymal one); (c) down-regulation of FA oxydatoin in tubular epithelial cells [105–107]; and the activation of the renin–angiotensin–aldosterone system (SRAA) [108–110].

### Blocking the renin–angiotensin system to prevent fibrinogenesis

Activation of the SRAA is a key pathway for the development of chronic cardiovascular disease.

Angiotensin II (AngII) has been shown to induce cytokine secretion by tubular cells and promoting the accumulation of inflammatory cells in both the tubular and glomerular compartments [108]. The MD2/TLR4/MyD88 plays a pivotal role in mediating the proinflammatory effects of AngII [109]. Further damage to the kidney may arise from the activation of the coagulation cascade and leucocyte adhesion in microvessels [111]. Reciprocally, antagonization of AngII confers renal protection in a model of subtotal nephrectomy in rats [110]. Unsurprisingly, AngII has been widely utilized to enhance the onset of renal injury in animal models. Additionally, robust data suggest that AngII is a crucial contributor to the progression of renal fibrosis and chronic kidney disease via tissue inflammation and matrix protein deposition [109]. Conversely, some experimental work has suggested a deficit in SRAA activity, contributing to vasoplegia during distributive shock [112]. AngII has been investigated to restore the arterial pressure in patients on high doses of vasopressors in a recent RCT [113]. However, the long-term assessment, in particular concerning the occurrence of CKD in survivors, has not yet been carried out, particularly in patients treated for an extended period of time [114].

On the other hand, several observational data suggest a beneficial effect of blocking the SRAA in patients recovering from AKI. In a cohort of 611 patients with AKI during ICU stay and discharged alive from ICU, the presence of SRAA inhibitor at ICU discharged was associated with lower mortality with a propensity score-matched hazard ratio of 0.48 [0.27–0.85], *p* < 0.01) [115]. Similar results were observed in another large Canadian cohort, including 46,253 patients who suffered AKI during hospitalization. Blocking SRAA was associated with better outcomes at 2 years (HR = 0.85 [0.81–0.89], *p* < 0.01) but was not associated with ESRD or composite outcome composed by ESRD or sustained doubling of serum creatinine [116]. These results were not observed in an ancillary study of the AKIKI trial, which failed to evidence any beneficial association between SRAA blockers and 2-years outcomes in KDIGO3 survivors [117]. Of note, this study was likely to lack power. No increased risk of recurrent hospitalized AKI was observed after the new use of SRAA blockers suggesting that starting or resuming these medications is safe after AKI [118, 119].

### Follow-up

While the reassessment of patients 3 months after AKI is highly recommended by KDIGO guidelines [7], several studies have highlighted the fact that only a small proportion of patients ultimately benefit from this reassessment. Available data show that less than 30% of patients who suffered AKI during hospitalization are reassessed within the first year after discharge, including patients with CKD or pre-existing diabetes [120, 121], despite the current recommendation by nephrologists [122]. However, such a follow-up seems to impact the outcome through the optimization of treatments, detection, and prevention of cardiovascular diseases and prevention of new episodes of AKI. In an Ontario population-based cohort study, 3,877 patients who suffered AKI treated by renal replacement therapy and discharged alive from the hospital were evaluated depending on the completion of a follow-up consultation [123]. A visit with a nephrologist within 90 days after discharge was associated with a 24% decrease in mortality after 2 years of follow-up. However, with the increase in hospitalization rates complicated by AKI, the general application of reassessment may exceed existing capacity of nephrology programs. Given the poor outcome of AKI survivors, RCT and prospective observational studies, as the ongoing French PREDICT multicenter study [124], are needed to determine which subpopulations of patients would benefit most from these interventions.

## Perspectives on AKI research: an incredible playground in terms of epidemiology, basic science, and translational research

In recent years, due to the generalization of big databases, artificial intelligence (AI) techniques have been increasingly crucial in critical care. AKI is not exempt from the application of AI techniques, in particular, to predict AKI occurrence or aggravation [125–129]. A deep learning model developed on electronic health records from 703,782 adult patients could predict 55.8% of all episodes of AKI, 90.2% of all AKI required dialysis, with a lead time of up to 48 h and a ratio of 2 false alerts for every real alert [125]. However, the major limitation of these models is that the prediction of AKI is derived from variations in SCr, which remains an imperfect marker for renal function [130].

To date, the search for new biological (plasmatic or urinary NGAL, KIM-1, Cystatin C, TIMP-2, IGFBP7) or non-biological (intra-renal Doppler flow indices) marker of kidney injury represent an essential part of the literature with contradictory results. Rather than helping in the diagnosis of AKI, they can be useful in predicting the most severe forms of AKI [131] or detect kidney injury in patients not meeting the current definition of AKI (i.e. so-called sub-clinical AKI). If both RCTs AKIKI and IDEAL-ICU did not demonstrate any survival benefit according to the time to onset of RRT for all patients with stage 3 AKI, the high mortality rate observed in patients who underwent RRT later justifies the need to identify persistent AKI [98, 99]. In a multicenter international prospective observation study, Hoste et al. had identified for the first time a new urinary biomarker, the C–C motif chemokine ligand 14 (CCL_14_), with good discrimination (AUC = 0.83 [0.78–0.87]) [132]. If the discovery of CCL_14_ as a predictor of persistent AKI is not the first one to suggest the role of monocytes/macrophages in the pathophysiology of AKI, especially in sepsis [133], it offers the opportunity to identify new approaches of AKI therapy. Moreover, the promise of early intervention able to improve renal outcome in infraclinic AKI must be encouraged by the development of biomarkers research.

The impact of strategies of RRT on renal recovery remains poorly understood and should be explored. Finally, strategies to prevent long-term development of both chronic and cardiovascular diseases require full attention to limits the AKI “scar”.

Finally, behind therapeutic innovations, next years will bring out new endpoints that will allow us to better define endpoints of interest in the setting of AKI (Table 1), better report the prevalence of AKI/CKD/ESRD and overall survival, and improve our tools to measure actual real-time GFR, functional renal reserve and kidney damage.

**Table 1:** Classical and potential endpoint used to evaluate efficacy of AKI procedure

	Classical outcome	Potential outcome
Prevention	AKI prevalence	Real time GFR
	Change of biomarker	
Clinical management	ICU death	Time free of RRT
	Dialysis dependency at discharge	AKI as competitive event to death/complicatoin
		Recovery of renal function
		Functional renal reserve
Follow-up	Long-term survival	Measure of GFR
	End-stage kidney disease	Patient-related quality of life
	CKD	

*AKI* acute kidney injury, *GFR* glomerular filtration rate, *ICU* intensive care unit, *RRT* renal replacement therapy, *CKD* chronic kidney disease

## Conclusion

AKI is highly prevalent among ICU patients and has been associated with short- and long-term outcomes. Several therapeutic strategies can either prevent or mitigate the consequences of AKI. Future research should now identify sub-phenotypes of AKI with different response to available treatments, tools for earlier and better recognition of kidney damage and renal function and innovative therapeutic strategies with the ultimate goal of improving patient-centered outcomes.

## Data Availability

Not applicable.

## Abbreviations

AKI: Acute kidney injury
ICU: Intensive care unit
CKD: Chronic kidney disease
RRT: Renal replacement therapy
ESRD: End-stage renal disease
ACR: Albumine-to-creatinine ratio
RBF: Renal blood flow
mABP: Mean arterial blood pressure
GFR: Glomerular filtration rate
RCT: Randomized control trial
PGC1α-NAD: PPAR gamma coactivator 1 alpha nicotinamide adenine dinucleotide
FA: Fatty acid
CRRT: Continuous renal replacement therapy
IHD: Intermittent hemodialysis
ATN: Acute tubular necrosis
SRAA: Renin–angiotensin–aldosterone system
AngII: Angiotensin II
CCL_14_: C–C motif chemokine ligand 14
